# The neutrophil-lymphocyte ratio to predict poor prognosis of critical acute myocardial infarction patients: a retrospective cohort study

**DOI:** 10.11613/BM.2023.010702

**Published:** 2022-12-15

**Authors:** Wenhui Wang, Linlin Liu, Zhongping Ning, Lin Che, Xinming Li

**Affiliations:** 1 Tongji University School of Medicine, Shanghai, China; 2 Department of Cardiology, Shanghai University of Medicine & Health Sciences Affiliated Zhoupu Hospital, China; 3 Department of Cardiology, Shanghai Tongji Hospital, Tongji University School of Medicine, China; 4 Shanghai Pudong New Area Center for Disease Control and Prevention, Shanghai, China

**Keywords:** acute myocardial infarction, neutrophil-lymphocyte ratio, mortality, inflammation

## Abstract

**Introduction:**

Inflammation is closely related to adverse outcomes of acute myocardial infarction (AMI). This study aimed to evaluate whether neutrophil-lymphocyte ratio (NLR) can predict poor prognosis of critical AMI patients.

**Materials and methods:**

We designed a retrospective cohort study and extracted AMI patients from the “Medical Information Mart for Intensive Care-III” database. The primary outcome was 1-year all-cause mortality. The secondary outcomes were 90-day and in-hospital all-cause mortalities, and acute kidney injury (AKI) incidence. The optimal cut-offs of NLR were picked by X-tile software according to the 1-year mortality and patient groups were created: low-NLR (< 4.8), high-NLR (4.8 - 21.1), and very high-NLR (> 21.1). Cox and modified Poisson regression models were used to evaluate the effect of NLR on outcomes in critically AMI patients.

**Results:**

Finally, 782 critical AMI patients were enrolled in this study, and the 1-year mortality was 32% (249/782). The high- and very high-NLR groups had a higher incidence of outcomes than the low-NLR group (*P* < 0.05). The multivariate regression analyses found that the high- and very high-NLR groups had a higher risk of 1-year mortality (Hazard ratio (HR) = 1.59, 95% CI: 1.12 to 2.24, P = 0.009 and HR = 1.73, 95% CI: 1.09 to 2.73, P = 0.020), 90-day mortality (HR = 1.69, 95% CI: 1.13 to 2.54, P = 0.011 and HR = 1.90, 95% CI: 1.13 to 3.20, P = 0.016), in-hospital mortality (Relative risk (RR) = 1.77, 95% CI: 1.14 to 2.74, P = 0.010 and RR = 2.10, 95% CI: 1.23 to 3.58, P = 0.007), and AKI incidence (RR = 1.44, 95% CI: 1.06 to 1.95, P = 0.018 and RR = 1.34, 95% CI: 0.87 to 2.07, P = 0.180) compared with low-NLR group. NLR retained stable predictive ability in sensitivity analyses.

**Conclusion:**

Baseline NLR is an independent risk factor for 1-year mortality, 90-day mortality, in-hospital mortality, and AKI incidence in AMI patients.

## Introduction

Acute myocardial infarction (AMI) is one of the significant causes of death with a high incidence (*1*). The estimated annual incidence of AMI in the United States is 650,000 (*2*). The rupture of atherosclerotic plaques is the most common cause of AMI, and infiltration of inflammatory cells can be found in ruptured plaques (*3*). Cardiomyocyte death and inflammatory response triggers are observed in AMI patients (*4*). Studies have shown that inflammation indicators, such as C-reactive protein (CRP), systemic immune-inflammation index (SII), and neutrophil-lymphocyte ratio (NLR), are clearly related to myocardial infarction (*5*-*7*).

The NLR, first proposed by Zahorec in 2001, is an inflammation-related index calculated from complete blood count (CBC) (*8*). It is reported to be associated with atherosclerosis, tumours, and Behcet’s disease (*9*-*12*). High NLR appeared to be associated with mortality and major adverse cardiovascular events (MACE) in patients with AMI (*13*). High NLR in patients with non-ST-elevation myocardial infarction (NSTEMI) was reported to be associated with higher mortality and major adverse cardiovascular events (MACE), while Cho *et al.* recommended NLR combined with anaemia as an independent risk factor in patients with ST-elevation myocardial infarction (STEMI) (*13*-*15*). In addition, NLR also has predictive power in AMI patients with cardiogenic shock and after reperfusion therapy (*16*, *17*). In contrast, NLR had better predictive power for mortality after NSTEMI compared with neutrophil-monocyte ratio (NMR), platelet-lymphocyte ratio (PLR), and lymphocyte-monocyte ratio (LMR) (*13*). However, the population and outcomes of NLR reported in AMI patients were highly heterogeneous, and the definitions of high/low NLR vary widely among studies (*10*).

This retrospective cohort study was designed to evaluate whether NLR can predict the poor prognosis of critical patients with AMI, and to explore optimal cut-off values of NLR in AMI.

## Materials and methods

### Methods

Hospitalization information of AMI patients admitted to Beth Israel Deaconess Medical Center from 2001 to 2012 was extracted from the Medical Information Mart for Intensive Care-III (MIMIC) database, while the death information was from the Social Security database. The MIMIC-III database was approved by the Institutional Review Boards (IRB) of the Massachusetts Institute of Technology (MIT, Cambridge, USA) and the Beth Israel Deaconess Medical Center (BIDMC). The authors passed the “Protecting Human Research Participants” exam and obtained permission to access the dataset (authorization code: 43259734). Since the information in the database was anonymous, informed consent was exempt. The study complied with the ethical standards laid down in declaration of Helsinki.

We extracted patients’ clinical information such as the essential information, laboratory indicators, sequential organ failure assessment (SOFA) score, SII, comorbidities, medications, and reperfusion therapy. Essential information included age, gender, body mass index (BMI), smoke status, and survival status. Laboratory indicators included white blood cell count (WBC), neutrophil percentage (N%), lymphocyte percentage (L%), haemoglobin (Hb), haematocrit (Hct), red cell distribution width (RDW), platelet count (PLT), and serum creatinine (SCr). Comorbidities included hypertension (HT), diabetes mellitus (DM), hyperlipidaemia, heart failure (HF), shock, prior myocardial infarction (priorMI), and atrial fibrillation (AF). Medications included taking angiotensin Converting Enzyme Inhibitor (ACEI), β-blocker, aspirin, statin, and clopidogrel. Additionally, obesity was defined as BMI ≥ 30 kg/m^2^ (*18*). The neutrophil-lymphocyte ratio was calculated as N%/L%. Because NLR was the primary object of our study, patients with missing N% or L% data were excluded directly. In addition, multivariate imputation was used for variables with less than 30% missing values.

### Inclusion and exclusion criteria

Among more than 50,000 critical patients in the database, patients who met the following criteria were included: (*1*) intensive care unit (ICU) patients; (*2*) ICU stay ≥ 24 hours; (*3*) discharge diagnosis of AMI according to the International Classification of Diseases (ICD) - Ninth Revision code between 410.00 and 410.92 (*19*). The exclusion criteria were: (*1*) < 18 or > 90 years; (*2*) missing CBC data; (*3*) patients diagnosed with trauma, leukaemia or lymphoma, chronic inflammatory, malignant disease, or recent blood transfusion. Only the records of the first ICU admission were included for repeated admissions.

### Definition of outcomes in AMI patients

Mortalities in our study were defined as all-cause mortalities. The primary outcome was mortality within one year since admission (hereafter referred to as 1-year mortality). In contrast, secondary outcomes were mortality within 90 days since admission (hereafter referred to as 90-day mortality), in-hospital mortality, and AKI incidence. Acute kidney injury was defined as increased SCr concentrations to ≥ 1.5 times baseline within the previous seven days according to the Kidney Disease: Improving Global Outcomes (KDIGO) criteria (*20*).

### Statistical analysis

The optimal cut-off values of NLR were picked by X-tile software (Yale University, New Haven, USA) according to the 1-year mortality of critical AMI patients (Supplementary Figure 1) (*21*). Moreover, patients were divided into three groups: low-NLR (< 4.8), high-NLR (4.8-21.1) and very high-NLR (> 21.1).

The Shapiro-Wilks method was used to test the normality of continuous variables. Continuous variables that conform to normal distribution were expressed as mean ± standard deviation (SD) and analysed by Student’s *t*-test; otherwise, they were expressed as median (interquartile range, IQR) and analysed by Wilcoxon rank-sum. Categorical variables were expressed by the number of the observations divided with the total number of subjects within the group (N/total), and Chi-square test or Fisher’s exact probability method was used to analyse the difference in constituent ratios. *Post hoc* analyses were performed with Bonferroni adjustment. Gender, age, and variables with P < 0.05 in difference analysis were included as confounding variables in multivariate Cox and modified Poisson regression models with NLR. Variance inflation factor (VIF) was calculated between confounding variables using R packages “performance” and “see”, and a VIF > 5 indicated the presence of multicollinearity that is detrimental to the regression model (*22*). In order to verify the robustness of the results, a series of sensitivity analyses were performed.

All statistical tests were two-sided with α = 0.05 and considered statistically significant with P < 0.05. Researcher A performed analyses using R software, version 4.0.3 (R Core Team, Vienna, Austria), and researcher B used IBM SPSS Statistics for Windows, version 26.0 (IBM Corporation, Armonk, United States), respectively.

## Results

### Baseline characteristics

**Figure 1:**
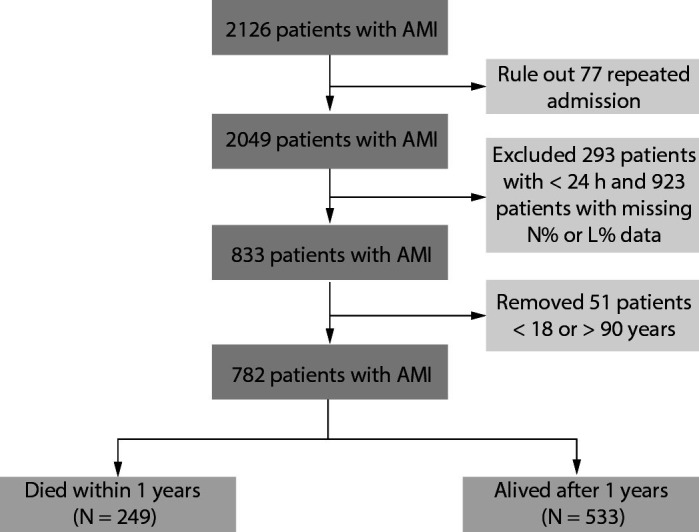
Flow chart of patients enrolled in the study. AMI - acute myocardial infarction. N% - neutrophil percentage. L% - lymphocyte percentage.

Among 782 critical AMI patients enrolled, the median (range) age was 68 (19-89) years, and 35% were female (272/782). The 1-year mortality of admission was 32% (95% confidence interval (CI): 29 - 35%; 249/782), of which 193 died within 90 days and 138 died in the hospital. The flow chart of enrolled patients is shown in Figure 1 (Figure 1).

Baseline characteristics are shown in Table 1. Compared with the low-NLR group, high- and very high-NLR groups had higher WBC, N%, and SCr concentrations and lower L% in laboratory results; and the very high-NLR group had higher RDW levels than low- and high-NLR groups. High- and very high-NLR groups had a higher incidence of outcomes than the low-NLR group (P < 0.05).

**Table 1:** Baseline characteristics of low-, high- and very high-NLR groups

**Characteristic**	**low-NLR (<4.8),** **N = 195**	**high-NLR (4.8-21.1),** **N = 503**	**very high-NLR (>21.1),** **N=84**	**P**
Female, N (%)	76 (41)	165 (33)	31 (37)	0.280
Age, years	66 (34-89)	69 (19-89)	69 (23-88)	0.414
Smokers, N (%)	23 (12)	60 (12)	11 (13)	0.949
Obesity, N (%)	49 (25)	137 (27)	19 (23)	0.621
WBC, x 10^9^/L	11.0 (8.1-13.9)	13.0 (10.2-16.4)*	17.4 (12.9-21.1)^*†^	< 0.001
N%, %	69 (63-74)	84 (80-88)*	91 (88-94)^*†^	< 0.001
L%, %	21 (18-27)	9 (7-12)*	3 (2-4)^*†^	< 0.001
Hb, g/L	115 (97-130)	116 (101-132)	115 (100-129)	0.499
Hct, L/L	0.340 (0.294-0.378)	0.345 (0.298-0.391)	0.335 (0.305-0.384)	0.495
RDW, %	14 (13-15)	14 (13-15)	14 (14-17)^*†^	< 0.001
PLT, x 10^9^/L	216 (165-277)	227 (170-276)	225 (181-281)	0.255
SCr, μmol/L	80 (71 -115)	97 (71-133)*	106 (80-177)*	< 0.001
SOFA	3 (1-6)	4 (2-7)*	6 (3-9)^*†^	< 0.001
SII	656 (402-980)	1988 (1363-3076)*	6873 (5402-9378)^*†^	< 0.001
**Outcomes, N (%)**				
AKI incidence	40 (21)	146 (29)	26 (31)	0.053
In-hospital mortality	19 (10)	92 (18)*	27 (32)^*†^	< 0.001
90-day mortality	29 (15)	130 (26)*	34 (44)^*†^	< 0.001
1-year mortality	41 (21)	169 (34)*	39 (46)*	< 0.001
**Comorbidities, N (%)**				
HT	100 (51)	218 (43)	31 (37)	0.053
DM	56 (29)	152 (30)	25 (30)	0.927
Hyperlipidaemia	37 (19)	114 (23)	12 (14)	0.164
HF	76 (39)	251 (50)*	47 (56)*	0.010
Shock	37 (19)	131 (26)	21 (25)	0.145
PriorMI	11 (6)	31 (6)	4 (5)	0.868
AF	55 (28)	166 (33)	28 (33)	0.452
**Medications, N (%)**				
ACEI	111/195 (57)	286 (57)	37 (44)	0.082
β-blocker	151/195 (77)	383 (76)	55 (65)	0.081
Aspirin	119/195 (61)	279 (55)	47 (56)	0.405
Statin	89/195 (46)	222 (44)	33 (39)	0.614
Clopidogrel	56/195 (29)	178 (35)	21 (25)	0.070
Reperfusion	137/195 (70)	334 (66)	38 (45)^*†^	< 0.001
Age is presented as median (range). Continuous data are presented as median (interquartile range). Categorical data are presented as the number of the observations divided with the total number of subjects within the group. *Statistically significant difference between this group and the low-NLR group. ^†^ Statistically significant difference between this group and the high-NLR group. NLR - Neutrophil-lymphocyte ratio. WBC - White blood cell count. N% - Neutrophil percentage. L% - Lymphocyte percentage. Hb - Haemoglobin. Hct - Haematocrit. RDW - Red cell distribution width. PLT – Platelet count. SCr - Serum creatinine. SOFA - Sequential Organ Failure Assessment. SII - Systemic immune-inflammation index. AKI - Acute kidney injury. HT - Hypertension. DM - Diabetes mellitus. HF - Heart failure. priorMI - Prior myocardial infarction. AF - Atrial fibrillation. ACEI - Angiotensin Converting Enzyme Inhibitor.				

### Predictive ability of NLR for primary outcome

**Figure 2:**
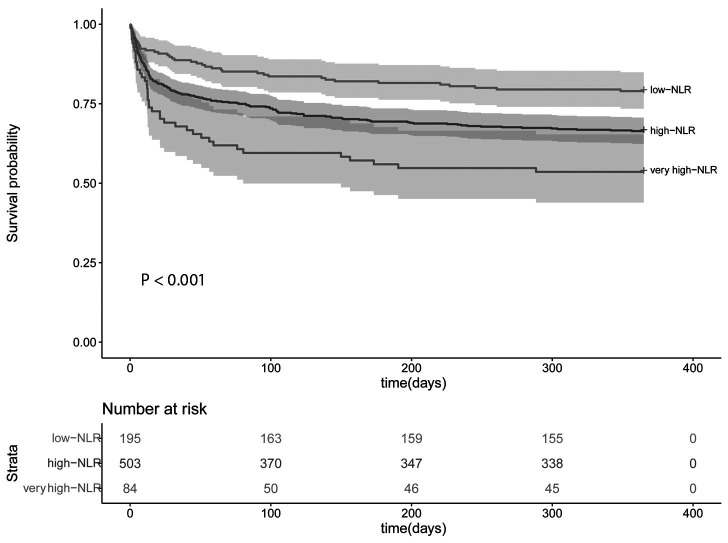
1-year Kaplan-Meier Survival curve of three NLR Groups. NLR - Neutrophil-lymphocyte ratio.

The 1-year mortality of low-, high- and very high-NLR groups was compared by log-rank and univariate Cox regression analysis, and Kaplan-Meier (KM) survival curve was drawn (Figure 2). The results showed statistical differences among three groups (log-rank P < 0.001), and high NLR appeared to contribute to mortality. Because of the baseline imbalance between the three groups (Table 1), multivariate Cox regression was used to control for bias caused by confounding variables.

**Figure 3:**
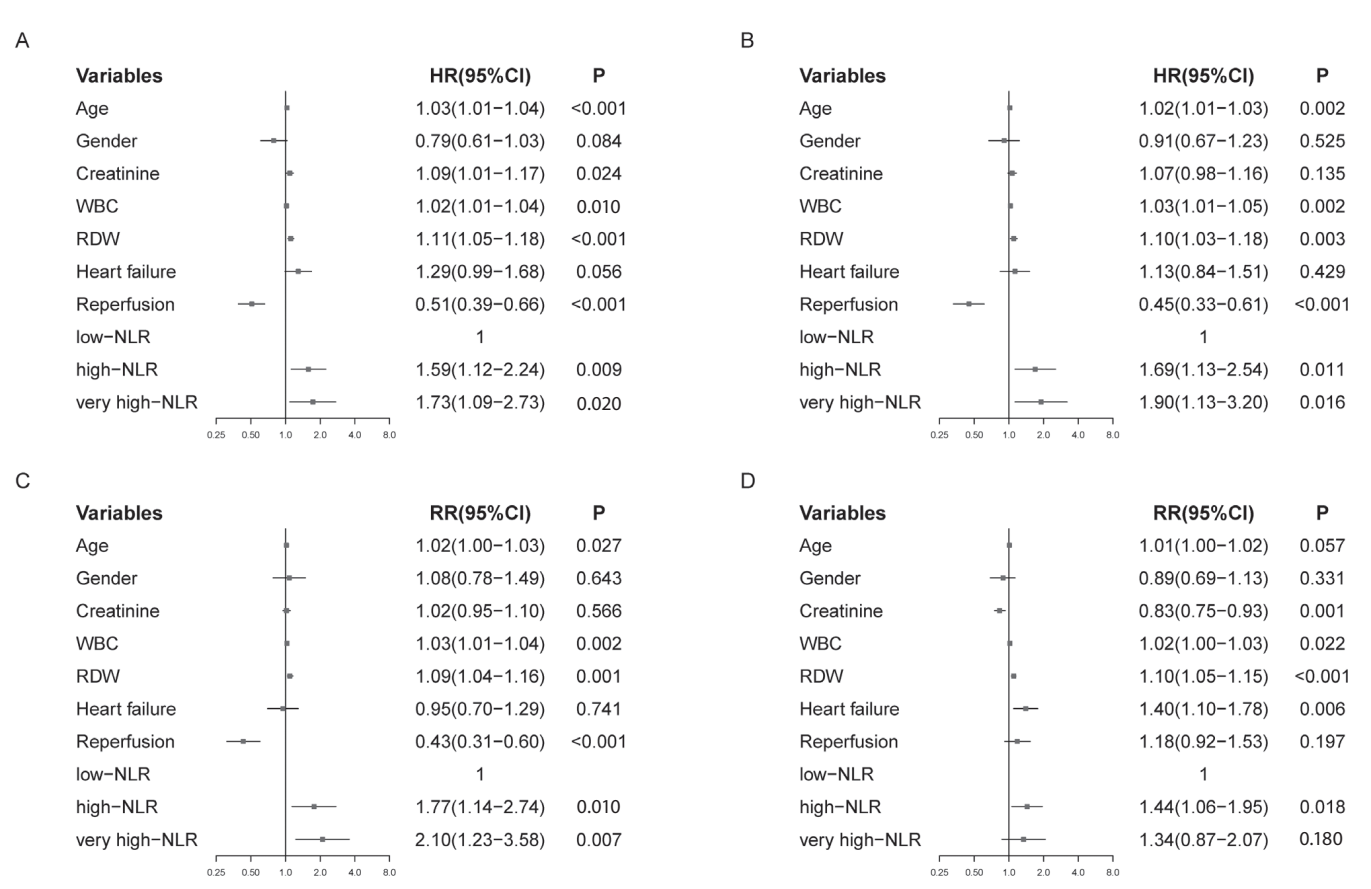
Multivariate regression analyses for the primary and secondary outcomes. A. Multivariate regression analyses for 1-year mortality; B. Multivariate regression analyses for 90-day mortality; C. Multivariate regression analyses for In-hospital mortality; D. Multivariate regression analyses for AKI incidence. NLR - Neutrophil-lymphocyte ratio. HR - Hazard ratio. CI - Confidence interval. AKI - Acute kidney injury. RR - Relative risk. WBC - white blood cell count. RDW - red cell distribution width.

There was no multicollinearity between NLR and confounding variables as all VIFs were < 5 (Supplementary Figure 2). After adjusting for gender, age, SCr, WBC, RDW, HF, and reperfusion therapy, it was found that compared with the low-NLR group, the high- and very high-NLR groups had a higher risk of 1-year mortality (Hazard ratio (HR) = 1.59, 95% CI: 1.12 to 2.24, P = 0.009 and HR = 1.73, 95% CI: 1.09 to 2.73, P = 0.020, respectively) (Table 2, Figure 3A).

**Table 2:** Cox regression analysis of NLR on 1-year and 90-day mortality

		**unadjusted**			adjusted*		
**Outcomes**	**Groups**	**HR**	**95% CI**	**P**	**HR**	**95% CI**	**P**
1-year mortality	low-NLR						
	high-NLR	1.74	1.24-2.45	0.001	1.59	1.12-2.24	0.009
	very high-NLR	2.69	1.74-4.18	< 0.001	1.73	1.09-2.73	0.020
90-day mortality	low-NLR						
	high-NLR	1.85	1.24-2.77	0.003	1.69	1.13-2.54	0.011
	very high-NLR	3.17	1.93-5.20	< 0.001	1.90	1.13-3.20	0.016
*Adjusted for gender, age, serum creatinine, white blood cell count, red cell distribution width, and heart failure and reperfusion therapy. The low-NLR was considered the control group. NLR - Neutrophil-lymphocyte ratio. HR - Hazard ratio. CI - Confidence interval.							

### Predictive ability of NLR for secondary outcomes

The univariate analysis found that both high- and very high-NLR groups had higher rates of 90-day mortality, in-hospital mortality, and AKI incidence than the low-NLR group. After adjusting for gender, age, SCr, WBC, RDW, HF, and reperfusion therapy, it was found that high- and very high-NLR groups had higher 90-day mortalities and in-hospital mortalities, and the high-NLR group had a higher AKI incidence than the low-NLR group (Table 2-3, Figure 3B-D). No significant difference was found in AKI incidence between low-NLR and very high-NLR groups (P > 0.05) (Table 3, Figure 3D).

**Table 3:** Modified Poisson regression analysis of NLR on in-hospital mortality and AKI incidence

		**unadjusted**			adjusted*		
**Outcomes**	**Groups**	**RR**	**95%CI**	**P**	**RR**	**95%CI**	**P**
In-hospital mortality	low-NLR						
	high-NLR	1.88	1.18-2.99	0.008	1.77	1.14-2.74	0.010
	very high-NLR	3.30	1.95-5.60	< 0.001	2.10	1.23-3.58	0.007
AKI incidence	low-NLR						
	high-NLR	1.42	1.04-1.93	0.027	1.44	1.06-1.95	0.018
	very high-NLR	1.51	0.99-2.30	0.056	1.34	0.87-2.07	0.180
*Adjusted for gender, age, serum creatinine, white blood cell count, red cell distribution width, and heart failure and reperfusion therapy. The low-NLR was considered the control group. NLR - Neutrophil-lymphocyte ratio. AKI - Acute kidney injury. RR - Relative risk. CI - Confidence interval.							

### Sensitivity analysis

**Figure 4:**
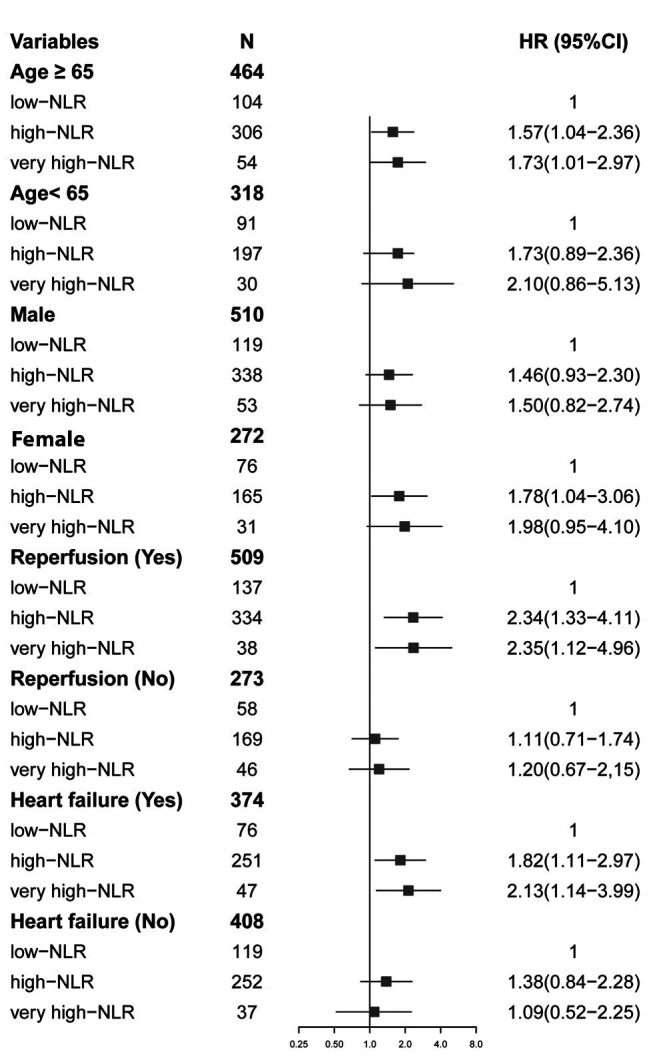
Subgroup analysis of three NLR Groups. NLR - Neutrophil-lymphocyte ratio. HR - Hazard ratio. CI - Confidence interval.

Sensitivity analyses were performed further to verify the predictive ability of NLR in different populations. First, NLR’s best dichotomous cut-off value was 8, which was obtained from X-tile. Cox regression and modified Poisson regression were used to analyse the relationship between NLR and outcomes in AMI patients. It was found that patients with NLR ≥ 8 had higher 1-year mortality (HR = 1.37, 95%CI: 1.05 to 1.78, P = 0.021), 90-day mortality (HR = 1.51, 95%CI: 1.11 to 2.05, P = 0.008) and in-hospital mortality (RR = 1.62, 95%CI: 1.17 to 2.24, P = 0.004) than that with NLR < 8, but no significant difference was found in AKI incidence (RR = 1.27, 95%CI: 1.00 to 1.60, P = 0.052) between two groups (Supplementary Table 1-2). Additionally, subgroup analyses were performed. The results showed that NLR retained stable predictive ability in critical AMI patients aged ≥ 65 years, with reperfusion therapy and with HF (Figure 4).

## Discussion

This study identified that baseline NLR was an independent risk factor for adverse prognostic events in critically patients with AMI and provided optimal cut-off values of NLR for risk stratification. Moreover, we found that patients in high- and very-high NLR groups experienced higher risks for death within one year with 1.59‐ to 1.73‐fold higher hazards compared with patients in the low-NLR group. The clinicians can use the results of this study for quick risk stratification of AMI patients when flipping through the CBC results.

The neutrophil-lymphocyte ratio ranged from 0.78-3.53 in healthy adults, and there is currently no well-accepted reference value for NLR in most diseases (*23*). Previous studies of NLR mostly performed dichotomization of data. The neutrophil-lymphocyte ratio > 4 was associated with worse overall survival in patients with solid tumours, while it was > 6.11 in Corona virus disease (*11*, *23*, *24*). Heart failure patients with elevated NLR (> 2.1 - 7.6) suffered from a worse prognosis (*25*). In AMI patients, the cut-off value was > 3.30 - 8.16 (which was highly variable), and in sepsis patients, it was as high as the value of 10 (*26*-*28*). However, dichotomizing continuous variables, such as taking the median, will lead to a considerable loss of information and a higher risk of false positives (*29*). At this time, multi-classification can combine clinical needs well and reduce information loss (*30*). Therefore, this study provided two reference values of 4.8 and 21.1 for NLR in critical AMI patients by X-tile software, which were validated to be specific, robust, and of practical value.

A series of subsequent statistical analyses showed that patients with higher inflammatory indicators of WBC and SII tend to have higher levels of NLR, suggesting that NLR could reflect the body’s inflammatory state, which was consistent with previous studies (*7*). We also found that the proportion of reperfusion therapy decreased with the increase in NLR. It might result from multiple factors, such as onset time of chest pain, age, patient willingness, and so on. Multivariate analysis suggested that both high- and very high-NLR were independent predictors of poor prognosis for critical AMI patients. A similar result was found by Liu *et al.* that NLR ≥ 3.17 was significantly associated with higher mortality and incidence of MACE in AMI patients (*14*). However, our study classified NLR in a more detailed way, allowing clinicians to carry out more precise risk stratification. Patients with high- and very high-NLR had a higher risk of death within one year, and the same trends were observed in the 90-day and in-hospital mortality. However, for the incidence of in-hospital AKI, patients with high-NLR were at higher risk than those with low-NLR; the very high-NLR group had a higher incidence of AKI compared with the low- and high-NLR groups, but the differences were not statistically significant (P > 0.05), probably caused by the limited sample size of this group. Previous studies have suggested that advanced age, chronic kidney disease, and HF are related to inflammatory response status and associated with higher mortality of AMI patients (*31*, *32*). Yan *et al.* found that NLR was a potential independent marker of mortality in elderly patients with AMI, which was consistent with our subgroup analysis (*33*). Physicians need to be vigilant in AMI patients with high- and very high-NLR, especially those with advanced age, higher SCr and WBC concentrations, HF, and those without reperfusion therapy.

Our study has two limitations. First, this was a single-center retrospective study with a predominantly Caucasian population (491/782, 63%). So, there might be some selection bias. Second, some prognostic factors such as CRP, troponin, infarct location, and left ventricular function have been shown to correlate with the prognosis of AMI, but these were not included in our study (*34*). Therefore, the findings should better be further verified by prospective, multicenter studies.

In conlusion, the neutrophil-lymphocyte ratio is an independent prognostic factor of 1-year mortality, 90-day mortality, in-hospital mortality, and AKI incidence in critical AMI patients. More attention should be paid to AMI patients with NLR ≥ 4.8, especially those with advanced age, higher SCr and WBC concentration, HF, and those without reperfusion therapy.

## Data Availability

The data supporting this study are available from Medical Information Mart for Intensive Care III database, but there are some restrictions to the availability of these data, which were used under license for the current study, and so are not publicly available. Data are available from the authors upon reasonable request.
